# Disparities in Cancer Clinical Trials: An Analysis of Comprehensive Cancer Control Plans

**Published:** 2009-09-15

**Authors:** Tisha Moniek Felder, Gabriela D. Pena, Bridget F Chapital

**Affiliations:** Center for Health Promotion and Prevention Research, University of Texas School of Public Health, University of Texas Health Science Center-Houston; Baylor College of Medicine, Houston, Texas; Baylor College of Medicine, Houston, Texas

## Abstract

**Introduction:**

Disparities in enrollment of adults in cancer clinical trials are well documented, but little is known about the attention given to this topic in comprehensive cancer control (CCC) plans. We assessed the extent to which CCC plans address disparities in clinical trials and whether jurisdictions whose plans address disparities also mandate third-party reimbursement for clinical trial participation.

**Methods:**

We analyzed 57 CCC plans identified from Cancer PLANET (Plan, Link, Act, Network with Evidence-based Tools) and Cancerplan.org from April through December 2007. We searched plans for general and disparity-specific content regarding clinical trials and analyzed the content for emergent themes. We assessed frequencies of themes, patterns between themes, and patterns between themes and laws. We reviewed third-party reimbursement laws, as of September 2007, as recorded by the National Cancer Institute's State Cancer Legislative Database.

**Results:**

Fifty-five (96%) CCC plans had content regarding clinical trials. Of the 39 (71%) plans that specifically addressed disparities, 13 (33%) were in a state with laws mandating third-party reimbursement. Increasing participation and education, awareness, and outreach were the most common themes identified.

**Conclusion:**

Although many CCC plans address disparities in clinical trials, few of those plans are in jurisdictions that have third-party reimbursement laws.

## Introduction

Knowledge gained through clinical trials has been critical to preventing, diagnosing, and treating cancer. However, not all cancer patients benefit equally from these improvements. Nationally, 3% to 5% of adult cancer patients participate in cancer clinical trials (1). Racial/ethnic minorities have represented less than 15% of all adult participants in National Cancer Institute (NCI) treatment trials (2), and a review of Food and Drug Administration (FDA) cancer trials found that adults aged 65 years or older represented barely one-third of clinical trial participants, even though they account for approximately 60% of cancer cases in adults (3). Other adult populations, such as those living in rural areas (4), those who are low income (5), or those without health insurance or third-party reimbursement for clinical trials (5), are also less likely to participate.

Participants in clinical trials should reflect the populations affected by the particular diagnosis (6). Underrepresentation in clinical trials results in disparity in favor of participants who benefit from those trials. From a scientific perspective, diverse representation is necessary to test for differences in outcomes and to ensure the safety of therapies across a range of biological and genetic characteristics (6). From an ethical viewpoint, distributive justice requires that both the benefits and risks associated with clinical trial research be fairly distributed among those who are potentially affected (7). The goals in addressing disparities in clinical trial participation should be not only attaining representative participation among all groups of potential cancer patients but also fair opportunity to be aware of and participate in clinical trials without financial considerations.

The National Institutes of Health (NIH) and FDA mandate the inclusion of women and minorities in clinical trials (8,9). However, researchers have documented barriers to participating in cancer clinical trials. Barriers include lack of awareness (10), lack of being invited or recruited to participate (11), eligibility criteria that may exclude certain groups (12), fear or mistrust of the medical and scientific community, and cultural barriers (eg, language, beliefs, attitudes) (11). In health care systems, physicians may not refer older patients to clinical trials because of concern about toxicity or side effects from the treatment (1) and may exclude patients because of their own prejudices (13). At a policy level, barriers may include the lack of state mandates for insurance coverage for clinical trials and the lack of appropriate enforcement and oversight of existing mandates (6).

Because barriers to and disparities in cancer clinical trial participation exist on multiple levels, addressing these issues requires a comprehensive approach. One such approach is reflected in the comprehensive cancer control (CCC) plan for each state, tribe, territory, or other jurisdiction. The CCC plan approach helps prevent duplication of effort, reduce gaps in interventions, and enhance existing programs by coordinating and integrating community resources within a given state, tribe, territory, or other jurisdiction (14). Through the CCC planning process, various stakeholders, including those representing health departments, national organizations, universities, and local organizations, are brought into a statewide coalition to agree to local priorities and goals and to promote the plan among the stakeholders. These plans provide a basic, initial indicator of how each jurisdiction proposes to address their particular burden of cancer, including addressing disparities in cancer clinical trials. We describe the extent to which existing CCC plans address disparities in clinical trials and, of the plans that do address the issue, which ones come from jurisdictions with third-party reimbursement laws.

## Methods

### Data sources and collection

From April through December 2007, we analyzed CCC plans available on the Web sites Cancer Control PLANET (Plan, Link, Act, Network with Evidence-based Tools) (cancercontrolplanet.cancer.gov/state_plans.jsp) and CancerPlan.org (www.cancerplan.org). Sponsored by federal and national organizations, Cancer Control PLANET is a Web portal designed to provide public health professionals with access to data and resources to design, implement, and evaluate evidence-based cancer control programs and plans. The Web portal includes links to state-specific cancer incidence and mortality rates, research-tested intervention products, and CCC plans. Similarly, the Cancerplan.org Web site — a collaborative effort of the Centers for Disease Control and Prevention (CDC), American Cancer Society, and NCI — was developed to help states and other jurisdictions develop, implement, and evaluate their respective CCC plans by sharing best practices, resources, and tools. As a follow-up step, we conducted a keyword search via the Internet search engine Google in February 2008 to ensure that we had retrieved the most recent CCC plans in our initial search.

From Cancer Control PLANET and CancerPlan.org, we identified 57 CCC plans; the Internet search identified updates to 6 of them. All CCC plans were in Adobe Acrobat Portable Document Format (.pdf) (Adobe Systems, Inc, San Jose, California), which allowed us to search each plan using keywords. Our search strategy was a 2-step process. In step 1, we identified the CCC plans that addressed clinical trials by searching for "clinical trial(s)," "randomized trial(s)," "clinical research," "treatment trial(s)," "investigational therapy," or "experimental treatment." This search strategy was based on terms used in a previous study (15). We identified 55 of the initial 57 CCC plans as including goals, objectives, strategies, or actions relevant to clinical trials. From these 55 CCC plans, we extracted the relevant text verbatim. We did not extract text from a plan's introduction, background, or other purely narrative sections.

In step 2, we identified the plans that specifically addressed disparities related to clinical trials. We developed a disparity-specific keyword search strategy based on terms from the existing literature (12) and a randomly selected sample of 5 CCC plans. Four plans from tribes or territories were deemed automatically relevant because of their specific focus on underrepresented populations. We searched the remaining 51 plans identified by step 1 for the terms "disparities/disparity," "underserved," "high-risk," "underrepresented," "culture/cultural," "language," "linguistic," "low-income," "diverse/diversity," "underprivileged," "rural," "elderly," "older adults," "aged," "disadvantaged," "uninsured," "race/racial," "ethnic," "minority/minorities," "black/African American," "Hispanic," "American Indian/Native American," "Asian," or "tribe/tribal," and extracted the relevant text verbatim.

We used NCI's State Cancer Legislative Database (SCLD) to determine which states had mandatory coverage laws for third-party reimbursement (16). The SCLD is a searchable database of synthesized information from enacted state laws and resolutions that address cancer control topics.

### Analysis plan

We analyzed the extracted text using an inductive and iterative approach to identify emergent themes (17). Each author independently reviewed the extracted texts and categorized the data into themes. We then compared proposed themes and used a constant comparison process to define and refine the themes. Final decisions were reached by the consensus of all authors.

We conducted a frequency count of each theme, analyzed the relationship patterns between themes, and examined the patterns between themes and third-party coverage laws. We read the goals, strategies, and objectives sections of the plans that addressed clinical trial disparities to see whether they focused on specific cancers or specific populations, and then recorded our results.

## Results

### Summary of CCC plans

The CCC plans' starting dates ranged from 2001 to 2007, and the ending dates ranged from 2004 to 2011. All but 2 of the 57 plans proposed goals, objectives, strategies, or actions related to clinical trials in general. Among the 55 plans mentioning clinical trials, 39 (71%) specifically addressed disparities in participation. No pattern appeared to exist between the dates of the plans and whether they addressed the subject of disparities. Of the 55 jurisdictions represented by the plans, 20 (35%) had some type of third-party reimbursement law, which varied for different types of insurers and phases of clinical trials. Coverage for clinical trial participation was mandatory in 19 of the 20 jurisdictions, and 1 prohibited exclusion of coverage for clinical trials. Nine of the states without reimbursement laws but with CCC plans addressing disparities advocated for some level of insurance coverage or reimbursement for clinical trial participation from private or public insurers or both in their plans.

### Disparity-specific CCC plans

We derived 10 main themes from the 39 plans dealing with clinical trial disparities (Table 1) and an 11th "other" category for subjects addressed by only 1 plan. The other category included topics such as the need to expand clinical trial infrastructure, to develop continuing education resources on clinical trials, and to increase the participation of minority physicians in the conduct of clinical trials.

Table 2 presents a summary of the disparity-specific themes and third-party coverage laws in each of the 55 CCC plans that mentioned clinical trials. The Texas CCC plan included the most theme areas (n = 7), but Texas did not have a third-party coverage law. Delaware, Indiana, and Oklahoma each covered 5 theme areas, but only Delaware had a coverage law. More than half of the 39 CCC plans that specifically addressed clinical trial disparities included the themes "increasing participation" (n = 24) or "education, awareness, and outreach" for underrepresented groups (n = 20); 12 plans included both these themes. Among the 39 disparity-specific CCC plans, 13 were in states with a third-party reimbursement law. The "education, awareness, and outreach" theme and the "availability of trials" theme surfaced in all 4 tribal or territory CCC plans. The themes "culturally appropriate information," "financial support," and "funding" were the least frequently mentioned among the plans. No pattern appeared to exist between the dates of a plan and whether it mentioned a specific theme.

Among plans addressing the topic of disparities, some had a focus on specific cancers or underrepresented groups. Four plans mentioned a focus on a specific cancer: Arizona (prostate), California (lung, oral), New Hampshire (lung), and New Jersey (prostate). Table 3 highlights which plans mentioned a specific underrepresented group.

## Discussion

Nearly all of the CCC plans we identified addressed clinical trials in general, and most specifically addressed disparities in clinical trials. The themes most often addressed were increasing the number of underrepresented participants in clinical trials and education, awareness, and outreach. In general, themes that emerged were related to barriers that have been documented in the literature. For example, education, awareness, and outreach corresponds to the consistently reported barrier that many patients are unaware of clinical trials (10,12). The lack of available trials has been cited as a barrier, particularly among patients in rural communities (18). These correspondences suggest that CCC planners are aware of existing barriers and are proposing strategies to directly address those barriers.

Although not having health insurance coverage is often a barrier to participating in clinical trials (5,6), even those with insurance coverage sometimes forgo participating in clinical trials because they fear their health insurance will not reimburse them (19). One study found that even after reimbursement laws were in place, clinical trial participation significantly increased for NCI phase II cooperative group trials but not for phase III (20). Conversely, another study found no increase in participation but did report that insurance type was no longer a significant factor in determining whether patients would enroll in cancer trials following mandatory third-party reimbursement legislation (21). These studies highlight the fact that reimbursement laws are not the lone solution to increasing participation in clinical trials. The comprehensive efforts reflected in the 10 themes that emerged in our study are examples of steps that may play a major role in addressing disparities in clinical trial participation.

CCC plans are at different stages of development and implementation. However, some CCC coalitions are already taking concrete steps to address clinical trial disparities. The Cherokee Nation is working with the Oklahoma Society of Oncologists to facilitate access to clinical trials and provide information about available local trials on the CCC Web site (22). The Delaware Cancer Consortium has seen its state's insurance code and regulations amended to cover cancer clinical trial participation (23). Minnesota is using outreach programs and community health workers to educate and increase awareness of clinical trials among racial and ethnic minorities (24). These examples suggest that including disparities in clinical trials as a topic in CCC plans would provide support for CCC coalitions and other local stakeholders to act on it.

Our findings have a few limitations. First is our keyword search strategy. Though we believe our keyword terms are inclusive, we cannot assume that the remaining plans did not cover the themes we identified. Second, there was variability in CCC plan format, specifically with regard to level of specificity. It is likely that the more specific a plan was in describing its clinical trials and disparities in clinical trials goals, objectives, strategies, or actions, the more likely we were to capture it based on our keyword search. Nevertheless, many of the themes that emerged from the information extracted from the CCC plans were consistent with future recommendations from other sources (6,12). Third, the data used in this study were extracted from planning documents made available on the Internet. Therefore, we were only able to capture data that were posted online and not necessarily all existing data.

Increasing the participation of racial/ethnic and other underrepresented groups in clinical trials is a national research objective (25), but to our knowledge, our study is the first to examine the attention given to the issue of disparities in cancer clinical trials in CCC plans. CCC plans are a unique, publicly available resource that can serve as a basic indicator of how the cancer community prioritizes disparities in clinical trials nationally; they have been used in previous studies on tobacco control and human papillomavirus (26,27). Findings from this study serve as a call to action to members of CCC planning coalitions and consortia. As a first step, if they have not identified the specific populations that are not appropriately represented in their local clinical trials, they need to identify them and make it a priority to correct the balance of participation. The next step is to prioritize the incorporation of strategies for overcoming these disparities in their updated or revised CCC plan.

## Acknowledgments

We thank Dr Armin Weinberg and the Eliminating Disparities in Clinical Trial (EDICT) project for their support. We thank Ms Leslie Given for her expertise in CCC planning and valuable input. We thank Ms Karyn Popham and the members of the Behavioral Science Doctoral Seminar for reviewing and providing constructive feedback on this manuscript.

Ms Felder was funded by a predoctoral fellowship, University of Texas School of Public Health Cancer Education and Career Development Program — National Cancer Institute/NIH Grant no. 2R25CA57712. Ms Pena was funded by an unrestricted educational grant from Genentech, Inc.

## Figures and Tables

**Table 1:** Summary of Themes That Emerged From Disparity-Specific Comprehensive Cancer Control (CCC) Plans (N = 39), United States

Theme	No. of Plans	Definition	Jursidiction/CCC Plan Example
Participation	24	To increase the number or proportion of diverse participants in cancer clinical trials	District of Columbia: Increase the participation of eligible minority residents in cancer-related clinical trials by 15% by 2010
Education, awareness, and outreach	20	To provide general information about clinical trials to patients, the public, or other groups through various modes or channels	New Jersey: Educate the public regarding the purpose and importance of participating in clinical trials for cancer, with special emphasis on addressing the concerns of minority populations
Availability of trials	10	To increase the actual number or types of clinical trials for participation	New York: Increase the number of clinical trials focusing on cancer prevention and control in high-risk populations
Best practices	5	To use methods, processes, or techniques that are beneficial to diversifying clinical trial participation	Louisiana: Distribute information about the factors that have led to high clinical trial enrollment rates in an indigent, primarily African American population
Identifying barriers	5	To assess the specific factors that inhibit patients from participating in clinical trials	Arizona: Identify barriers that inhibit participation in clinical trials within minority populations
Partnerships and collaborations	5	To aim to develop relationships with organizations, institutions, or other groups	Oregon: Increase the enrollment of underserved populations in clinical trials by developing community-based partnerships that work with culturally diverse and underserved communities
Physician involvement	5	To engage physicians or providers as a strategy for educating or recruiting patients to clinical trials	California: Increase the awareness of community oncologists of the need for participation of diverse groups in clinical trials by encouraging those efforts in oncology associations
Financial support	4	To advocate for resources that aim to decrease the nonroutine costs associated with clinical trial participation for patients	Colorado: Develop interventions to alleviate the financial cost of participation in clinical trials studying treatment, supportive care, and quality of life for underserved and underinsured cancer patients
Funding	4	To advocate for broad-level resources to address nonroutine costs associated with clinical trial participation	New Mexico: Establish polices to ensure that funding is available for necessary follow-up care for those living in tribal communities who are screened for cancer through clinical trials and government-supported screening programs
Culturally appropriate information	3	To explicitly state the incorporation of culturally or linguistically specific information into the design or dissemination of clinical trials information	Indiana: Provide information about clinical trials in a culturally sensitive manner, including consent forms that are tailored to meet the patients' reading levels

**Table 2:** Mandatory Third-Party Reimbursement Laws and Number of Disparity-Specific Themes Covered in Comprehensive Cancer Control Plans, by Jurisdiction (n = 55), United States

**Jurisdiction**	Plan Date	Third-Party Reimbursement Law	Total Disparity-Specific Themes
**State or area**			
Alabama	2006-2010	No	0
Alaska	2005-2010	No	0
Arizona	2005-2007	Yes	4
Arkansas	2001-2005	No	0
California	2004-2010	Yes	4
Colorado	2005-2010	No	3
Connecticut	2005-2008	Yes	1
Delaware	2007-2011	Yes	5
District of Columbia	2005-2010	No	1
Florida	2003-2006	No	2
Georgia	NA	Yes	0
Hawaii	2004-2009	No	0
Idaho	2006-2010	No	1
Illinois	2005-2010	No	4
Indiana	2005-2008	No	5
Iowa	2006-2011	No	0
Kansas	2005	No	2
Kentucky	NA	No	0
Louisiana	2004-2009	Yes	1
Maine	2006-2010	Yes	0
Maryland	2004-2008	Yes	1
Massachusetts	2006-2011	Yes	0
Michigan	2007	No	2
Minnesota	2005-2010	No	3
Mississippi	2006-2011	No	0
Missouri	2004	Yes	2
Montana	2006-2011	No	0
Nebraska	2005	No	0
Nevada	2005	Yes	2
New Hampshire	2005	Yes	1
New Jersey	2002-2007	No	3
New Mexico	2007-2011	Yes	2
New York	2003-2010	No	3
North Carolina	2001-2006	Yes	0
North Dakota	2006-2010	No	1
Ohio	2006-2010	No	1
Oklahoma	2006-2010	No	5
Oregon	2005-2010	No	3
Pennsylvania	2003	No	0
Rhode Island	2007	Yes	0
South Carolina	2005-2010	No	3
South Dakota	2005-2010	No	2
Tennessee	2005-2008	Yes	0
Texas	2005	No	7
Utah	2006-2011	No	1
Vermont	2006-2010	Yes	0
Virginia	2001-2005	Yes	3
Washington	2004-2008	No	0
West Virginia	2007	Yes	1
Wisconsin	2005-2010	Yes	2
Wyoming	2006-2010	No	1
**Tribe or Territory**			
Alaska Native Tribal Health Consortium	2005-2010	No	3
Cherokee Nation	2005-2007	No	3
Northwest Portland Area Indian Health Board	2007	No	3
South Puget Intertribal Planning Agency	2005-2012	No	2

Abbreviation: NA, not available.

**Table 3:** Underrepresented Groups Mentioned in Disparity-Specific Comprehensive Cancer Control (CCC) Plans, United States

**Underrepresented Group**	**CCC Plan**
African American	Arizona Delaware Louisiana South Carolina
Alaska Natives	Alaska Native Tribal Health Consortium Northwest Portland Area Indian Health Board
American Indians	Cherokee Nation Northwest Portland Area Indian Health Board
Hispanics	Delaware
Low income	Maryland West Virginia
Tribal communities	New Mexico South Puget Intertribal Planning Agency
Uninsured or underinsured	Colorado Maryland West Virginia
